# A Unique Expression of Keratin 14 in a Subset of Trophoblast Cells

**DOI:** 10.1371/journal.pone.0139939

**Published:** 2015-10-02

**Authors:** Wassim Abou-Kheir, Assaad Eid, Rabih El-Merahbi, Rebecca Assaf, Georges Daoud

**Affiliations:** Department of Anatomy, Cell Biology and Physiological Sciences, Faculty of Medicine, American University of Beirut, Beirut, Lebanon; Brigham and Women's Hospital, UNITED STATES

## Abstract

The placenta, a transient organ in human, is essential for pregnancy maintenance and for fetal growth and development. Trophoblast and stromal cells are the main cell types present in human placenta. Trophoblast cells are present in different subtypes depending on their differentiation state and their temporal and spatial location during pregnancy. The stromal cells are of extraembryonic mesenchymal origin and are important for villous formation and maintenance. Interestingly, many pregnancy–related diseases are associated with defect in trophoblast differentiation and villous integrity. Therefore, it's crucial to specifically identify each type of placental cells using specific markers. Keratins (CK) are widely used as marker of epithelial cells, cancer origin identification and in some cases as marker of stem/progenitor cells. Vimentin is widely used as marker of mesenchymal cells. The aim of this study is to characterize the presence of different keratins in human trophoblast cells and vimentin in stromal cells. Using immunohistochemistry on term placental sections, our results show that vimentin is solely expressed in stromal-mesenchymal cells while keratins 5, 7, 8, 14 and 19 are expressed in trophoblast cells. Interestingly, all keratins tested, except for keratin 14, were evenly expressed in all trophoblast cells. Keratin 14 was expressed in a subset of CK7 positive cells. Moreover, the same results were obtained when using freshly isolated cytotrophoblast cells or BeWo cells. In conclusion, this study is a crucial step in the advancement of our knowledge in placental cell type identification and characterization.

## Introduction

The placenta plays a major role in the maintenance of pregnancy and in the development of the fetus. After fertilization, the first differentiation process in mammalian zygote is the formation of the trophectoderm layer that gives rise to the placenta and the inner cell mass (ICM), which forms the embryo proper. Interestingly, trophectoderm cells are polarized and have the characteristic of an epithelium while ICM blastomeres are devoid of polarity [1–3]. This epithelialization is associated with an increase in E-cadherin expression and activity [4–6] which is a major component of adherens junctions (AJ) present in most epithelial tissues [7]. Loss of E-cadherin expression affects AJ formation that in turn interferes with tight junction (TJ) formation in epithelia [8, 9]. These TJ in the trophectoderm layer are essential for the formation of the blastocoel cavity and for continuing embryonic development [10]. Therefore, the presence of these AJ and TJ confirms the epithelial phenotype of the trophectoderm layer and of all its subsequent trophoblast cell derivatives. Interestingly, trophoblast cells are also reported to express many members of the keratins family [11] that are largely used to identify epithelial cells [12, 13].

Keratins, previously known as cytokeratins, are forming parts of intermediate filaments and they provide mechanical and structural support to epithelial cells [14]. In addition, keratins are reported to play a role in different cellular functions including protection from apoptosis [15, 16], protection of liver cells against stress [17], regulation of cell size and protein synthesis during wound healing [18] and protection of placental barrier function [19, 20]. The sequencing of the human genome identified 54 different keratin genes classified into type I and type II and each type is subdivided into epithelial and hair keratins [21]. Keratins assemble in heterodimers to form intermediate filaments using type I and type II proteins. Their pattern of expression depends on the epithelial cell type and the state of differentiation of these cells [13]. For example, CK8/CK18 are widely expressed in simple epithelia such as the liver, acinar cells of the pancreas, intestinal cells, pseudostratified epithelia (e.g. respiratory) and in complex epithelia (e.g. glandular) [13]. Moreover, CK8/C18 are the first keratins to appear during embryogenesis, as early as pre-implantation stage [22]. In the same manner, CK7/CK19 are expressed in some simple epithelia and are called secondary keratins to CK8/CK18. Furthermore, CK20 is expressed and almost restricted to intestinal epithelial cells [23, 24]. Interestingly, different keratins were reported to be present in human placenta. *Muhlhauser et al* [25] showed an expression of keratins 7, 8, 13, 18 and 19 in villous and extravillous trophoblast cells. Keratins 8, 17, 18 and 19 are reported to be expressed in endovascular trophoblast cells [26]. Moreover, keratin 7 is used as marker of trophoblast cells during cytotrophoblast isolation from human placenta [27, 28]. Interestingly, CK20 was only expressed in molar pregnancy (100 and 50% in total and partial mole respectively) while no expression was detected in normal placenta [29]. Finally, some keratins are used in tumor diagnosis of several carcinomas especially in metastatic cancer to identify the primary site of the tumor [13]. Therefore, the aim of this study is to identify the expression and localization of several keratins in human placenta, primary culture cytotrophoblast cells and the BeWo chorioncarcinoma cell line.

## Materials and Methods

### Human placental cytotrophoblast cells isolation and purity evaluation

Human term placentas were collected after normal vaginal delivery from 38–40 weeks pregnancies. This study was approved by the Institutional Review Board (IRB) of the American University of Beirut Medical Center (AUBMC) which waived the need for a consent form from the patients. Cytotrophoblast cells were isolated following the protocol described by Kliman et al [30] with minor modifications [27]. Briefly, fetal and maternal membranes were removed and tissue was cut into 1-inch cubes and washed extensively with 0.9% saline until blood-free specimens were obtained. Then, the tissue pieces were minced and subjected to 4 digestions containing trypsin (Sigma-Aldrich) and DNase (Roche life Science). After each digestion, the supernatant was collected and replaced with fresh digestion medium. The supernatant of the first digestion was discarded and the following ones were layered onto calf serum and centrifuged at 1215xg for 15 min. Pellets from the collected digestions were pooled and suspended in DMEM-HG (Dulbecco’s Modified Eagle Medium-High Glucose) containing PSN (2x) (Penicillin-Streptomycin-Neomycin), deposited on top of a discontinuous 5–70% (v/v) Percoll gradient and centrifuged at 507g for 25 min. Layers containing cytotrophoblast cells were collected, washed in DMEM-HG containing PSN (2x) and used for immunostaining using a cytospin chamber.

### Cell culture

BeWo cells (ATCC # CCL–98) were a gift from Dr Julie Lafond (University of Québec at Montréal, Canada); they were cultured in F-12K medium (Ham’s F-12-Kaighn’s), supplemented with 10% heat inactivated FBS and 1% penicillin/streptomycin.

### Immunohistochemistry, Immunofluorescence and confocal microscopy

#### Antibodies and Reagents

All information about the antibodies used in this study is listed in Table 1. Fluoro-gel II with Dapi was purchased from EMS (Electron Microscopy Sciences, PA).

**Table 1:** list of antibodies used in immunohistochemistry and immunofluorescence.

Antibodies	Company	Cat.no.	Lot. No.	Dilution	concentration	clone
Mouse monoclonal Anti-CK8	Covance	MMS-162P-250	D14AF00042	1/100	1mg/ml	1E8
mouse monoclonal anti-CK19	Millipore	MAB3238	NG1802538	1/500	1mg/ml	RCK108
rabbit polyclonal anti-CK14	Covance	PRB-155P-100	D12FF01492	1/500	1mg/ml	Poly19053
rabbit polyclonal anti-CK5	Covance	PRB-160P-100	10KC01862	1/100	1mg/ml	Poly19055
rabbit polyclonal anti-CK7	Abcam	Ab52870	467077	1/100	Discontinued	NA
mouse monoclonal anti-CK14	Abcam	Ab7800	GR35202	1/500	0.125mg/ml	LL002
mouse monoclonal anti-E-cadherin	Abcam	Ab1416	GR149062-3	1/100	NA	HECD–1
mouse monoclonal anti-Vimentin	Abcam	Ab8978	844525	1/100	1mg/ml	RV202
rabbit polyclonal anti-Vimentin	Santa Cruz	Sc-7557-R	F0111	1/100	200 μg/ml	C–20
Alexa 488 goat anti-mouse	Life technologies	A11029	1531669	1/200	2mg/ml	NA
Alexa 488 goat anti-rabbit	Life technologies	A11008	913909	1/200	2mg/ml	NA
Alexa 568 goat anti-mouse	Life technologies	A11004	927620	1/200	2mg/ml	NA
Alexa 568 goat anti-rabbit	Life technologies	A11011	NA	1/200	2mg/ml	NA

NA: not available

#### Immunohistochemistry

Term placental tissues were collected and fixed in 4% formalin overnight, rinsed in PBS, and transferred to 70% ethanol before standard processing to obtain paraffin-embedded sections. Unstained tissue sections were deparaffinized, and antigen retrieval was performed in a citrate buffer in a steamer at 100°C for 60 min followed by 30 min incubation at room temperature. Slides were treated with peroxidase block for 5 min and then blocking was performed with protein block from Leica biosystems (UK) for 5 min at room temperature. Primary antibody incubation was performed overnight at 4°C, followed by post primary block for 30 min. Slides were incubated then with Novolink polymer (Leica biosystems, UK) for 30 min followed by incubation with DAB chromogen prepared in Novolink DAB substrate buffer for 5 min. All slides were counterstained with hematoxylin.

#### Immunofluorescence

Double immunofluorescence was performed on tissue sections using the same protocol used for IHC with the following exceptions: The secondary antibodies were Alexa Fluor 488 conjugated goat anti‐mouse and goat ant-rabbit IgG and Alexa Fluor 568 conjugated goat anti‐rabbit and goat anti-mouse IgG. Slides were mounted with the anti-fade Fluoro-gel II with Dapi. For immunofluorescence of cell lines grown in vitro, adherent cells were fixed in 4% paraformaldehyde (PFA) in PBS for 10 min, followed by permeabilization with 0.5% Triton X‐100 in PBS for 3 min. Non‐specific sites were blocked by incubation in 3% BSA in PBS for 30 min. Cells were then incubated overnight at 4°C with the specified primary antibodies in the blocking buffer. Cells were washed, incubated with the respective secondary antibody (Alexa Fluor 488 or 568) in blocking buffer for 30 min at room temperature. Finally, slides were washed and mounted using the anti‐fade reagent Fluoro‐gel II with Dapi.

For cytospin cells, single-cell suspensions were washed twice with PBS, and 5 x 10^4^ cells were deposited on glass slides in PBS by centrifugation at 1,000 rpm for 2 minutes using a cytospin system from Thermo Shandon (Thermoscientific, PA). Cells were fixed and stained as described earlier.

Confocal microscopic analyses were performed using Zeiss LSM 710 confocal microscope and images were acquired and analyzed using the ZEN image software.

## Results

### Expression of Vimentin, E-cadherin and Keratin 7 in human term placental sections

**Fig 1:**
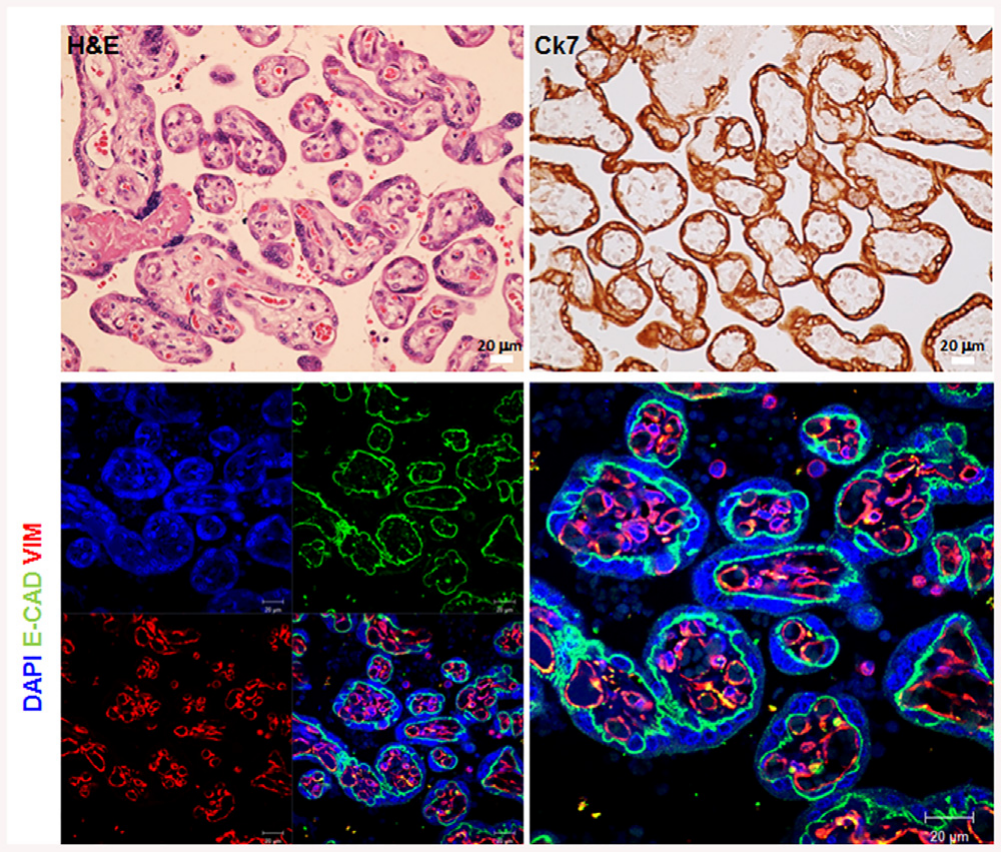
Basic histology of human term placenta. Paraffin-embedded sections of human term placenta are stained with H&E (A), CK7 (B) and E-Cad/VIM/Dapi (C). (D) is a higher magnification of (C). Representative images of at least 3 different placentas are shown.

During placental development, the villus is formed of two cell types. The outer layer is formed by epithelial cells called trophoblast cells and the villus core is formed of mesenchymal cells and blood vessels. E-cadherin and vimentin are widely used as marker of epithelial and mesenchymal cells respectively while CK7 is used as marker of trophoblast cells [27, 28]. We used these characteristics to identify and delineate the different cell types on placental cross sections. Therefore, sections were stained with H&E, E-cadherin, vimentin or CK7. The H&E staining showed a normal shape of villi containing blood vessels (Fig 1A) and surrounded by a layer of trophoblast cells as shown by CK7 staining (Fig 1B). As expected, our results showed a clear staining of E-cadherin in the trophoblast layer of villi while vimentin was specifically expressed in the mesodermal core of the villi (Fig 1C and 1D).

### Expression of cytokeratins in human term placentas

**Fig 2:**
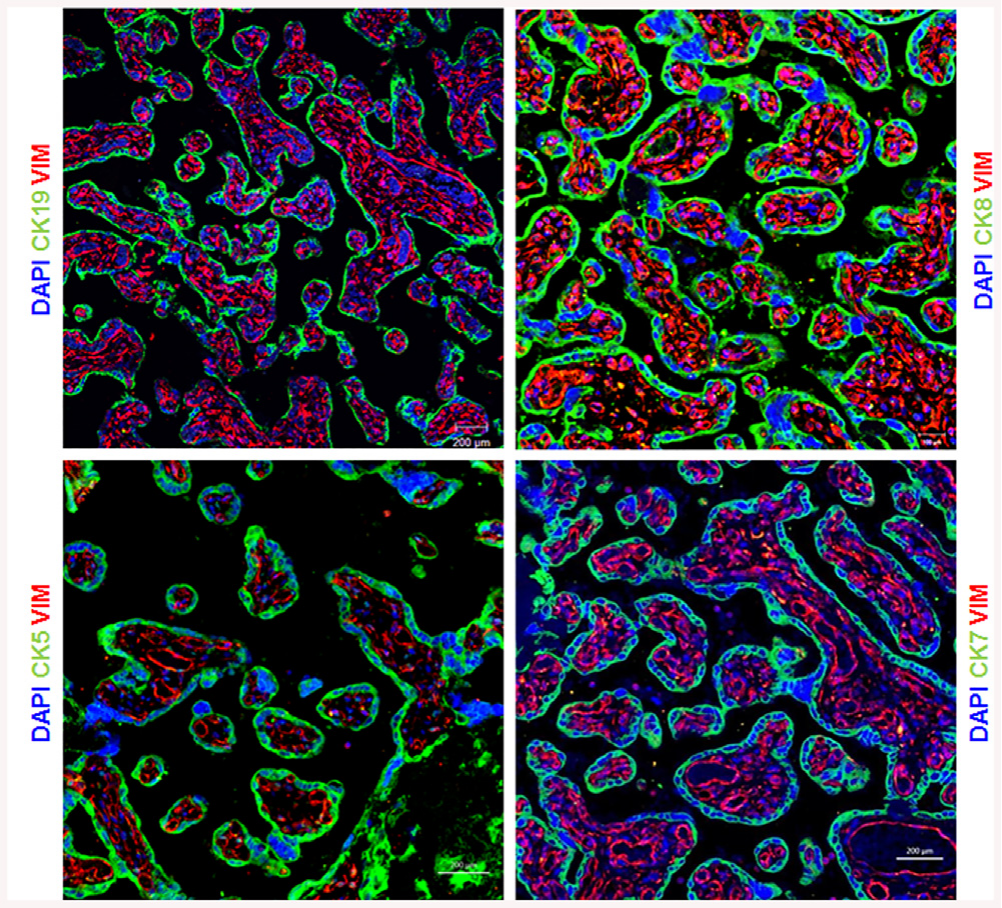
Trophoblasts are characterized by keratins expression. Paraffin-embedded sections of human term placenta are stained with Vimentin to mark the stromal cells in the villi, and with keratin 19 (CK19), keratin 8 (CK8), keratin 5 (CK5) and keratin 7 (CK7) to mark the trophoblast cells. Dapi is used to counterstain the nucleus. Representative images of 3 different placentas are shown.

The expression of different types of CK in human term placentas was evaluated using immunofluorescence staining. The results were the same in different placental sections and all control slides using rabbit or mouse IgG were negative. As shown in fig 2, CK5, 7, 8 and 19 were expressed in the trophoblast layer. Furthermore, a clear staining with vimentin of the mesenchymal core of villi is showed (Fig 2). Interestingly, no overlay expression between vimentin and any of the tested CKs is noted which further confirm the expression of CKs only in trophoblast cells.

**Fig 3:**
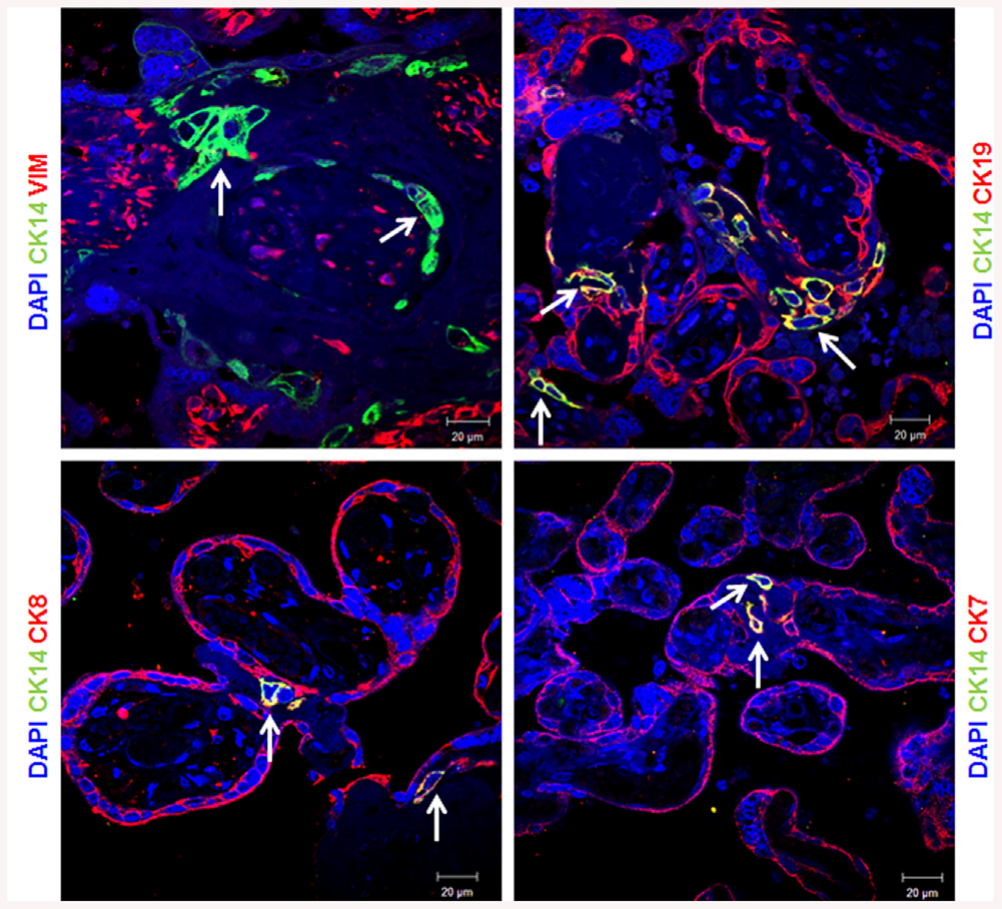
Expression pattern of keratin 14. Paraffin-embedded sections of human term placenta are stained with keratin 14 (CK14) along with Vimentin, keratin 19 (CK19), keratin 8 (CK8) and keratin 7 (CK7). Dapi is used to counterstain the nucleus. Representative images of 3 different placentas are shown. The arrows indicate the presence of CK14.

Surprisingly, CK14 expression is restricted to few cells in contrast to the expression of all CK tested that showed an equal distribution among the trophoblast layer (Figs 2 and 3). Moreover, CK14 expression was restricted to epithelial cells as all CK14 positive cells were negative for vimentin (Fig 3A). Furthermore, CK14 positive cells were also positive for CK7, CK8 and CK19 (Fig 3B and 3C) confirming their epithelial phenotype. These results suggest that CK14 might be a specific marker of a specific subpopulation of trophoblast cells.

### Expression of keratins in BeWo choriocarcinoma cell line

**Fig 4:**
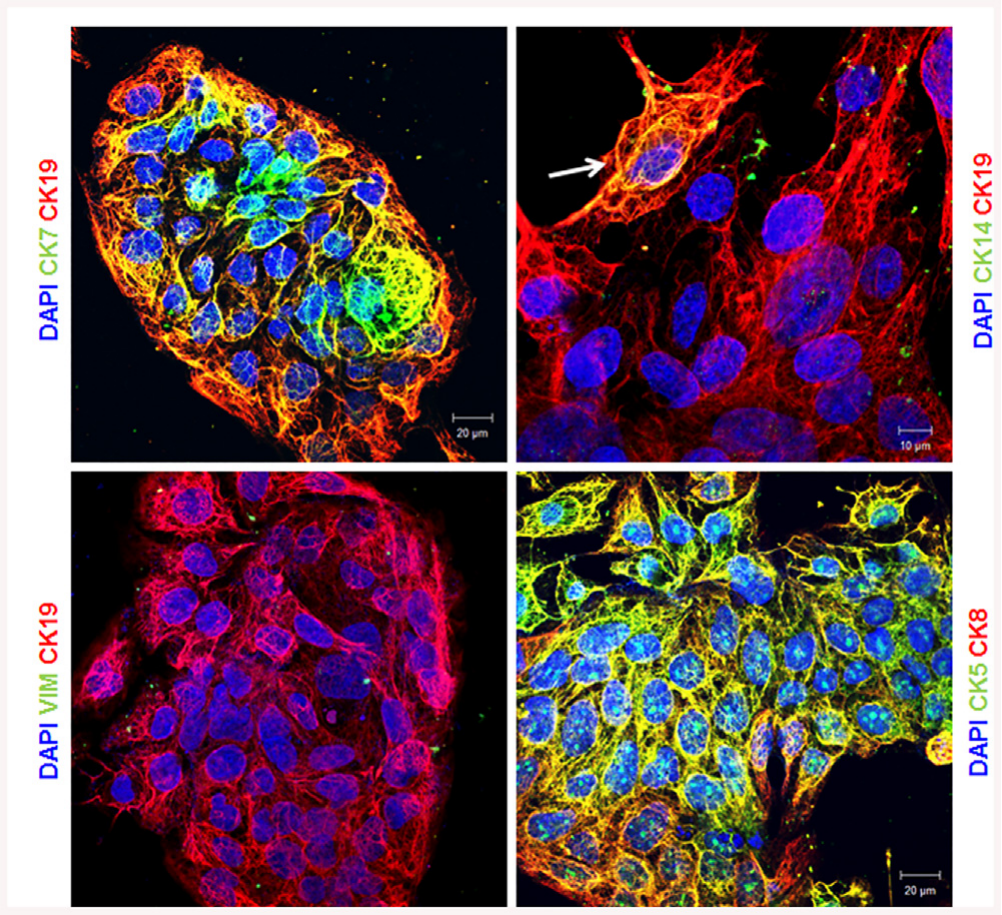
Keratins expression in BeWo cells. Representative immunofluorescent images of BeWo cells stained for the indicated antibodies and for Dapi are shown. The arrow indicates the presence of CK14. These experiments were repeated at least 3 times.

The expression of CKs was also evaluated in a choriocarcinoma cell line BeWo. These cells are derived from human placental trophoblast cells and therefore they are of epithelial origin. Moreover, they are largely used to study trophoblast differentiation and invasion [31–33]. As expected, BeWo cells expressed all type of keratins tested as shown for placental sections. They expressed high level of CK5, 7, 8 and 19 (Fig 4). Interestingly, the expression of CK14 in BeWo cells is restricted to a limited number of positive cells (Fig 4). This result is in line with our finding for placental sections.

### Expression of CK14 in freshly isolated cytotrophoblast cells

**Fig 5:**
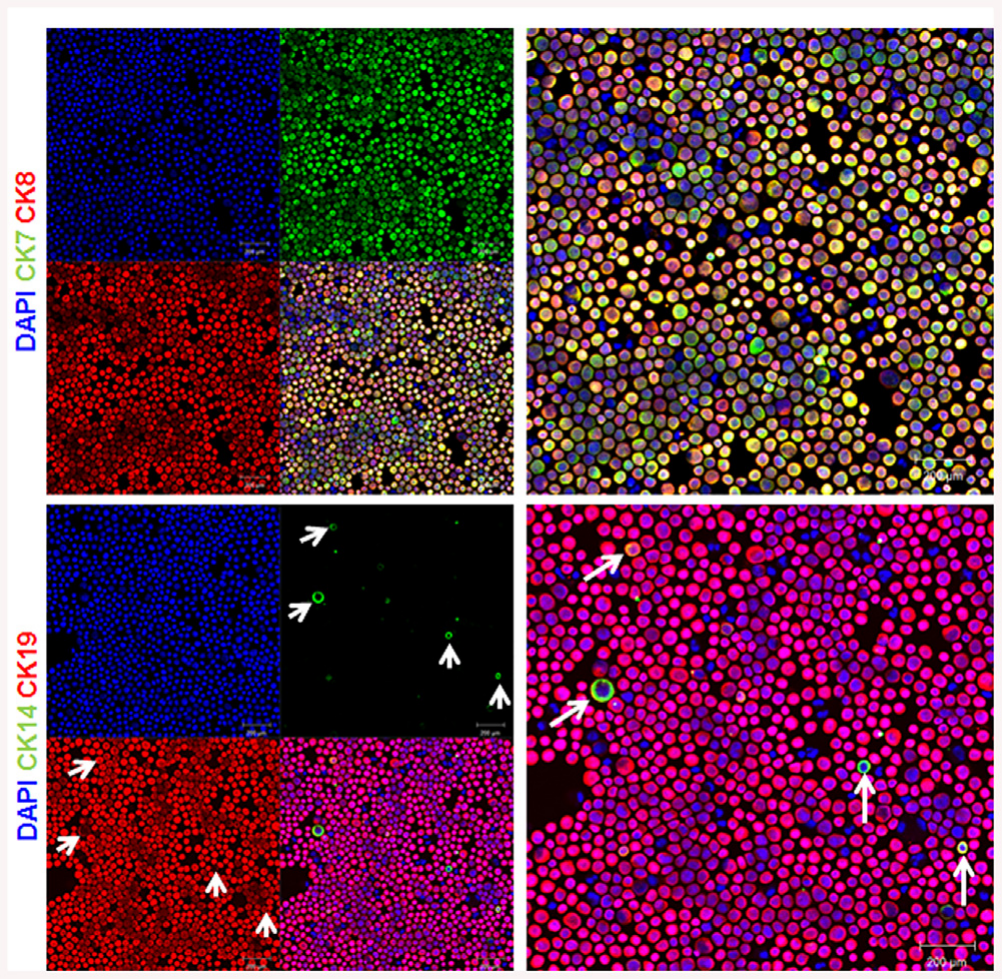
Keratins expression in primary trophoblast cells. Representative immunofluorescent images of cytospin preparations of primary trophoblast cells, isolated from human term placenta, stained for the indicated antibodies and for Dapi are shown. The arrows indicate the presence of CK14. These experiments were repeated at least 3 times.

**Fig 6:**
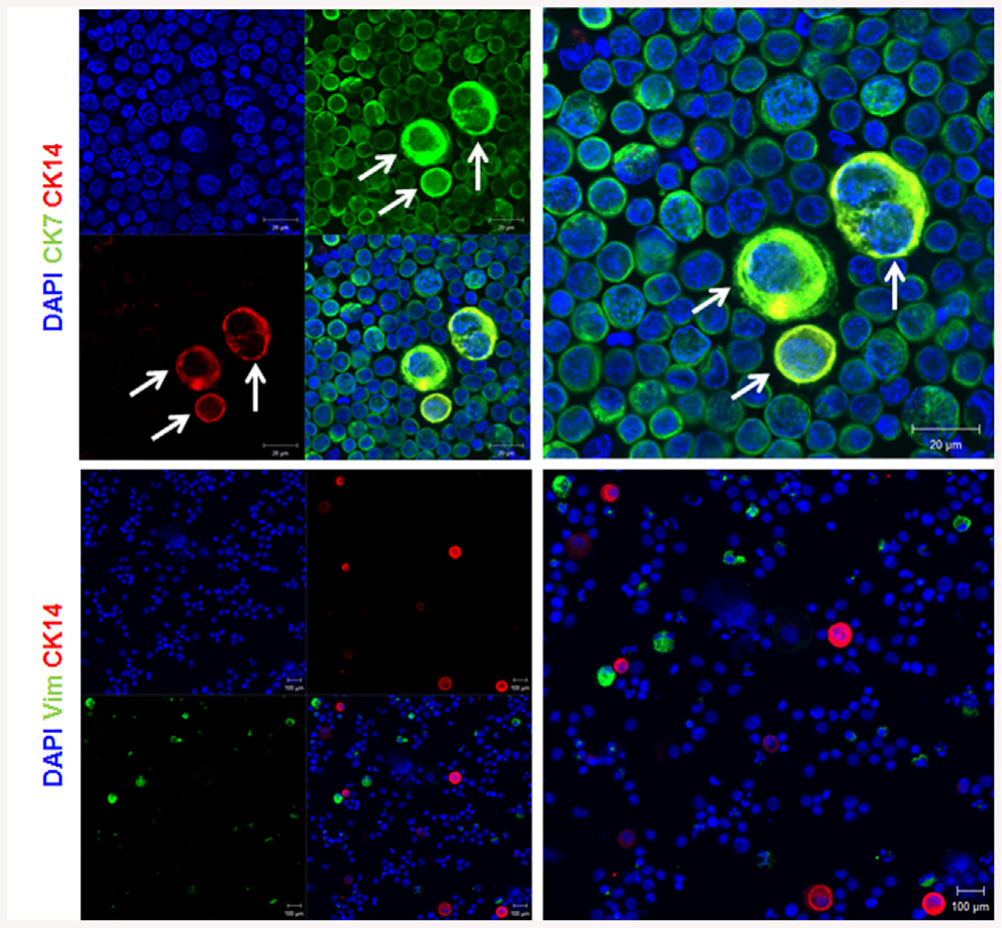
Expression of keratin 14 in primary trophoblast cells. Representative immunofluorescent images of cytospin preparations of primary trophoblast cells, isolated from human term placenta, stained for the indicated antibodies and for Dapi are shown. The arrows indicate the presence of CK14. These experiments were repeated at least 3 times.

Cytotrophoblast cells were freshly isolated from human term placentas of 38–40 weeks of pregnancy. The purity of cytotrophoblast cells was evaluated using CK7 as trophoblast marker [34]. Our results indicated that more than 95% of cells are positive for CK7 (Fig 5). Therefore, these cells were further screened for CK8, 19 and most importantly CK14. Fig 5 showed a clear expression of CK8 and CK19 in cytotrophoblast cells. Interestingly, CK14 expression was restricted to few cells as shown for BeWo cells and placental sections. The percentage of CK14 positive cells is less than 1% as shown in fig 5. In order to identify the origin of CK14 positive cells, freshly isolated cytotrophoblast cells were co-stained with either vimentin or CK7. Fig 6 shows that CK14 positive cells are also CK7 positive and vimentin negative confirming the trophoblast origin of these cells. Interestingly, it should be noted that CK14 positive cells are larger in size and bi-nucleated in some cases suggesting actively dividing cells (Fig 6).

## Discussion

Trophectoderm formation is the first differentiation event during human development and forms the wall of the blastocyst. This wall of trophoblast cells will ultimately participate along the extraembryonic mesoderm to form the placenta with all its cellular components [35]. Early during pregnancy, trophoblast cells are very important for normal implantation and for uterine wall invasion [36]. Interestingly, many pregnancy-related diseases are associated with a defect in trophoblast differentiation and function [37] and therefore, it is essential to distinguish between the different placental cell types when studying placental development and functions. Many studies have used different markers including vimentin, E-cadherin and cytokeratins to characterize the epithelial or mesenchymal origin of cells [38–40]. In this study, we showed a clear difference between the epithelial cells (E-cadherin positive) and stromal cells (vimentin positive). Previously, *Kohnen et al* showed that placental stromal cells specifically express vimentin at all stages of placental stem villi formation while all trophoblast cells were negative for vimentin as expected and in accordance with our results [41]. Therefore, vimentin can be used as marker of placental stromal cells. Interestingly, all epithelial cells stained positive for keratin 7, a marker of pan trophoblast cells [28, 42]. In this study, we report the expression of CK5, 7, 8, 14, and 19 in trophoblast cells. It should be noted that while many reports have reported an expression of different keratins in human placentas, none have reported an expression of CK14 in trophoblast cells [25, 29, 43]. In human placenta, keratins expression was reported to be up-regulated on the pathway to extravillous trophoblast cells [43]. Moreover, it has been reported that keratin expression varies among different trophoblast cells depending on their location and differentiation state [25]. Furthermore, *Haigh et al* reported an expression of cytokeratins 8 and 18 in a fibroblast cell strain isolated from placental explant [34]. All these results suggest that keratins are not only important for the cytoskeleton maintenance but also important molecules playing a role in cell function and differentiation.

Finally, keratins are widely used as markers of cancer origin and epithelial lineage including prostate, colon and breast [12, 38, 44]. Moreover, they are used as possible marker of stem cells population [13, 45]. In our study, we found that the expression of CK14 is limited to few cells in BeWo, freshly isolated cytotrophoblast cells and human placental sections suggesting that CK14 is only expressed in a subpopulation of trophoblast cells. CK17 was suggested as marker of intramural cytotrophoblast in human first trimester uteroplacental arteries [26] and K20 expression was shown to be expressed in gestational trophoblastic diseases such as complete mole [29]. Moreover, CK15 is used as marker of epidermal stem cells [45] while CK14 is used to identify the commitment of embryonic stem cells to an epidermal cell fate and their further differentiation into the basal layer of the epidermis [46]. In this study, we reported the expression of CK14 in a subpopulation of trophoblast cells while *Ahenkorah et al* reported a strong expression of CK14 in the amniotic epithelium with no expression in trophoblast cells [43]. Furthermore, Genbacev *et al* reported the establishment of human trophoblast progenitor cell lines from the chorionic membrane [47]. Therefore, it would be interesting to identify the origin of the CK14 positive trophoblast cells and if they are derivative of the CK14 positive amniotic epithelium. Finally, it would be interesting to identify the fate and characterize these CK14 positive trophoblast cells.

In conclusion, this study shows an expression of different keratins in human trophoblast cells in placental sections, freshly isolated cytotrophoblast cells and in BeWo cells. Interestingly, CK14 expression is expressed in a subpopulation of trophoblast cells. Since cytokeratins are used as markers of stem/progenitor cells, we suggest that this subpopulation of CK14 positive trophoblast cells might be a trophoblast progenitor/stem cells.
